# Development and validation of an alternative method for ergosterol determination in alder leaves using liquid-liquid extraction and LC-MS/MS after saponification

**DOI:** 10.1016/j.mex.2025.103495

**Published:** 2025-07-07

**Authors:** Björn Kusebauch, Stefan Bader, Steffen Carl

**Affiliations:** German Environment Agency (UBA), Section IV 2.5 - Trace Analysis, Artificial Ponds and Streams, Schichauweg 58 12307, Berlin, Germany

**Keywords:** Miniaturized sample preparation, Cryo-milling, Heat-reflux extraction, Semi-closed digestion unit, Fungal biomass, Leaf litter decomposition, Fungal biomarker

## Abstract

Ergosterol is widely used as proxy for the estimation of leaf litter associated fungal biomass. According to a common textbook method, ergosterol can be extracted from plant tissue with methanolic potassium hydroxide followed by purification with solid-phase extraction (SPE) and detection via high-performance liquid chromatography-ultraviolet (HPLC-UV). As an alternative, we developed a method using liquid-liquid extraction (LLE) of the methanolic extract with cyclohexane instead of time consuming and error prone SPE. For ergosterol detection, HPLC-UV was replaced by the more sensitive and selective liquid chromatography-tandem mass spectrometry (LC-MS/MS). The method was validated for alder leaves at 10 µg/g dw (LOQ) and 500 µg/g dw with mean recoveries between 95.1 and 100.2 % (relative standard deviations < 10 %) and has been successfully applied for the measurement of fungicide effects on leaf litter associated aquatic fungi.

• Selective and flexible analytical method for ergosterol quantification in alder leaves using LLE and LC-MS/MS instead of SPE and HPLC-UV
• Optional use of 7-dehydrocholesterol as internal standard to allow for compensation of volumetric variations during sample preparation and signal drift in LC-MS/MS
• Method validation for three mass transitions according to guidance document SANTE/2020/12830 including matrix effect evaluation and stability investigations

## Graphical abstract

**Figure:**
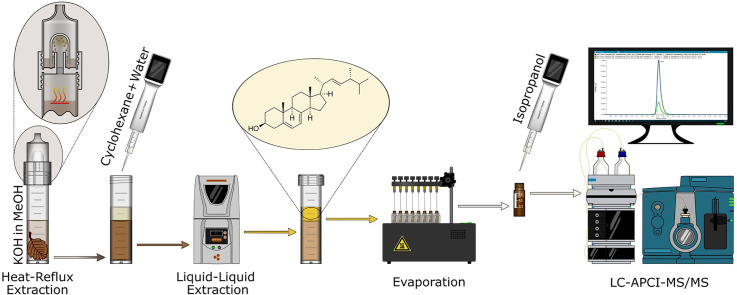

**Specifications table**

**Table:** 

**Subject area**	Environmental Science
**More specific subject area**	Biomarker analysis in fungal cell membranes
**Name of your method**	LC-MS/MS determination of ergosterol after saponification and liquid-liquid extraction
**Name and reference of original method**	M.O. Gessner, Ergosterol as a Measure of Fungal Biomass, in: F. Bärlocher, M.O. Gessner, M.A.S. Graça (Eds.), Methods to Study Litter Decomposition, Springer International Publishing, Cham, 2020: pp. 247–255. https://doi.org/10.1007/978–3–030–30515–4_27
**Resource availability**	Not applicable

## Background

Ergosterol is an important component of the fungal cell membrane with comparable functions to cholesterol in human cells [1,2]. For almost half a century, the analysis of ergosterol content has widely been used as proxy for the leaf litter associated fungal biomass [3]. A common textbook method for ergosterol determination in plant tissue by Gessner [4] relies on saponification with methanolic potassium hydroxide (KOH) at elevated temperature, solid-phase extraction (SPE) for sample purification and enrichment and subsequent detection with high-performance liquid chromatography-ultraviolet (HPLC-UV). Method details and specific precautionary measures when using SPE are discussed by Gessner et al. [5,6]. The method is applied by limnologists in ecological (e.g. [[7], [8], [9], [10]]) and chemical risk assessment studies (e.g. [[11], [12], [13]]) related to leaf litter decomposition in aquatic ecosystems.

Investigating the effects of azole fungicides on aquatic fungi [14], we were interested in treatment effects on fungal ergosterol synthesis. Azole fungicides inhibit the ergosterol biosynthesis by binding to the cytochrome P450 lanosterol 14α-demethylase and consequently cause a disruption of the fungal cell membrane and its vital functions [15]. Therefore, ergosterol serves as an interesting endpoint to evaluate the inhibitory effects of azole fungicides on fungi.

For ergosterol determination, we applied the saponification conditions, i.e. 8 g/L KOH in refluxing methanol, from Gessner [4] to allow for better comparison of results due to the frequent use of this method. Under these alkaline conditions, the total ergosterol content including ergosterol esters can be determined [6]. Gessner and Schmitt [5] could show that a KOH concentration of 8 g/L is sufficient for total ergosterol extraction.

The textbook method by Gessner [4] was adapted stepwise to the analytical equipment present in our lab. In the final method, semi-closed PFA (perfluoroalkoxy alkane) digestion units were used instead of pressure-resistant glass tubes to enhance lab safety. HPLC-UV was replaced by LC-MS/MS leading to an improved selectivity and sensitivity [16,17]. LC-MS/MS is more selective than HPLC-UV and thus less susceptible to interferences from co-eluting matrix components that might lead to false-positive results as shown by Headley et al. [16] and Verma et al. [18] for determination of ergosterol in different environmental matrices. Furthermore, 7-dehydrocholesterol was included as internal standard as suggested by Gessner and Newell [6]. The internal standard was used to compensate for potential volumetric variations during sample preparation and drift in mass spectrometric ionization response during analysis. With the increased sensitivity of LC-MS/MS we were also able to omit the SPE sample enrichment step. However, a sample clean-up was still necessary since the use of KOH is not compatible with mass spectrometers. For that, we used a single liquid-liquid extraction (LLE) with cyclohexane after addition of water to facilitate phase separation [19,20].

The final method relying on heat-reflux in methanolic KOH, subsequent partitioning of ergosterol into cyclohexane, evaporation and re-dissolving in isopropanol and detection via LC-MS/MS was validated according to guidance document SANTE/2020/12830 [21]. In total, the method including a previous version using screw-cap glass vessels instead of semi-closed digestion units [14] was successfully applied to > 300 alder leaf samples.

## Method details

### Chemicals and materials

Isopropanol (LC-MS hypergrade) was supplied by Merck (Darmstadt, Germany). Methanol (LC-MS grade), cyclohexane (for pesticide residue analysis) and potassium hydroxide (extra pure Ph. Eur.) were acquired from Chemsolute (Renningen, Germany). Formic acid (LC-MS grade) was purchased from VWR Chemicals (Darmstadt, Germany). Ultrapure water was freshly delivered per working day by a Milli-Q® Advantage A10 water purification system (Merck, Darmstadt, Germany). Ergosterol was obtained from Sigma-Aldrich (lot no. LRAD3304, purity 86.5 %; Steinheim, Germany), LGC (lot no. G1506005, purity 87.3 %; Wesel, Germany) and the European Directorate for the Quality of Medicines & HealthCare (catalogue code E1100000, batch 8, purity 100 %; Strasbourg, France). 7-dehydrocholesterol (purity 98.2 %) was acquired from Sigma-Aldrich (Steinheim, Germany). An 8 g/L solution of KOH in methanol was prepared by adding 8 g KOH to 1 L methanol followed by magnetic stirring (IKAMAG REC-G, IKA, Staufen, Germany) at room temperature for 1 hour at 400 rpm.

Since ergosterol is prone to photoconversion [[22], [23], [24]] and oxidative degradation [25,26], we highly recommend the use of amber glass ware and to purchase certified reference standards from different vendors. To ensure accurate preparation and stability of stock solutions before use (see section ‘*Stability of standard solutions and extracts*’), we compared stock solutions of each ergosterol reference standard during method development. Stock solutions of ergosterol (1 g/L) and 7-dehydrocholesterol (0.2 g/L) were prepared in amber glass vials by dissolving appropriate amounts of the reference compounds in isopropanol under consideration of purity and subsequent shaking for 30 min (2000 rpm, Multi Reax, Heidolph, Schwabach, Germany). Standard solutions containing 6 mg/L ergosterol for the preparation of calibration solutions and 20 mg/L for spiking of recovery samples at LOQ level of 10 µg/g dry weight (dw) were obtained from separate stock solutions by serial dilution with isopropanol. An internal standard solution of 0.6 mg/L 7-dehydrocholesterol in isopropanol was prepared from the respective stock solution. Ergosterol working solutions were obtained by diluting the 6 mg/L standard solution with isopropanol to concentrations of 1.8, 6, 12, 24, 60, 120, 300 and 600 ng/mL. Calibration solutions containing 1.5, 5, 10, 20, 50, 100, 250 and 500 ng/mL ergosterol as well as 100 ng/mL 7-dehydrocholesterol were prepared by spiking 1 mL of each ergosterol working solution with 0.2 mL of the 0.6 mg/L internal standard solution followed by vortexing and filtration through syringe filters (Rotilabo PP41 RC 0.20 µm, Roth, Karlsruhe, Germany). For the preparation of matrix-matched calibration solutions with concentrations of 5, 50 and 250 ng/mL, 120 µL aliquots of matrix blank extract were evaporated to dryness and re-dissolved in 1 mL of ergosterol working solutions with concentrations of 6, 12 and 300 ng/mL. After addition of 0.2 mL of the 0.6 mg/L internal standard solution, the matrix-matched calibration solutions were vortexed and filtered as described above. All solutions were kept in amber glass vials and stored at 4 °C until use.

For the preparation of blank and fortified samples for method validation, autoclaved black alder leaves (*Alnus glutinosa*) were used (leaves that were taken from alder tree branches and prepared as described in [27]). In order to reduce the natural background of ergosterol from leaf associated fungi (e.g. fungal endo- and epiphytes, plant pathogens), the leaf material was exposed to UV light prior to homogenization. For details see section ‘*Limit of detection and quantification*’.

### Sample homogenization

Prior to homogenization, alder leaf samples were freeze-dried overnight (> 16 h, Delta 2–24 LSC, Christ, Osterode, Germany). Samples with amounts up to 2 g were ground for 30 s at 25,000 rpm using a knife mill (Tube Mill control 100, IKA, Staufen, Germany) with disposable grinding chambers (MT 40, IKA, Staufen, Germany) after addition of one tablespoon of dry ice. For sample amounts larger than 1 g, the time required for cryo-milling might need to be increased to 2 × 30 s. The homogenized samples were placed in a fume hood for approximately 1 hour to allow for complete sublimation of carbon dioxide. To facilitate sample reanalysis, freeze-dried samples were further processed in aliquots.

### Sample preparation

For heat-reflux extraction, PFA digestion units (BaekDu, AHF analysentechnik, Tübingen, Germany) comprising a sample vessel and a condensing vessel with vapor trap were used. The semi-closed digestion units permit a pressure-free extraction under reflux conditions in contrast to traditionally used pressure-resistant glass tubes [4,16,18] or screw-cap test tubes [19,20].

For preparation of validation samples, 50 ± 1 mg of homogenized blank sample material were weighed into 50 mL sample vessels and spiked with 50 µL of the internal standard stock solution (0.2 g/L 7-dehydrocholesterol) except for double blanks (matrix blanks without internal standard). Subsequently, recovery samples were fortified with ergosterol at 10 µg/g dw (LOQ) and 500 µg/g dw by addition of 25 µL of standard solution (20 mg/L) and 25 µL of stock solution (1 g/L), respectively. In addition to blanks and double blanks, procedural blanks containing no matrix but internal standard were also prepared and analyzed.

As shown in section ’*Sample preparation robustness*’, the sample weight can vary between 10 and 100 mg without affecting the accuracy. Regardless of the sample amount, 10 mL methanolic potassium hydroxide solution (8 g/L) were added to each sample. Afterwards, the sample vessels were connected with the condensing vessels and the whole units were heated in a metal block thermostat (TM-130–36 Thermobil, Liebisch, Bielefeld, Germany) for 25 min at 110 °C. Subsequently, the digestion units were allowed to cool to room temperature for 15 min. The condensing vessels were unscrewed and remaining condensate was transferred into the sample vessels. 5 mL ultrapure water and 10 mL cyclohexane were added to each sample vessel with a positive displacement pipette (HandyStep Touch, Brand, Wertheim, Germany). Vessels were then closed with screw-caps and placed in a vertical shaker (1600 MiniG, Spex, Metuchen, USA) for 7.5 min at 1000 rpm followed by centrifugation (Sorvall Lynx 6000, Thermo Scientific, Langenselbold, Germany) at 2000 × *g* for 2 min. Multiple extraction of study samples demonstrated that a single extraction step with cyclohexane is sufficient to extract > 95 % of total ergosterol content.

In the literature, toluene [28], pentane [16,18,29], hexane [[30], [31], [32]], petrol ether [3,33], and cyclohexane [19,20] are applied successfully as partitioning solvents for ergosterol extraction. According to the Pfizer solvent selection guide [34], cyclohexane and toluene are the least toxic solvents among the aforementioned. The removal of toluene commonly requires reduced pressure and can be time consuming. Therefore, we used cyclohexane for sample clean-up.

100 µL of the cyclohexane phase were transferred into 2 mL HPLC vials (Macherey-Nagel, Düren, Germany) and fully evaporated with nitrogen (Multiplex valve depot, Liebisch, Bielefeld, Germany) at room temperature for approx. 5 min. If dilution or concentration is required, the volume of cyclohexane can be decreased to 50 µL and increased up to 400 µL, respectively (see section ’*Sample preparation robustness*’). The residues were re-dissolved in 1 mL of isopropanol followed by vortexing (Minishaker MS1, IKA, Staufen, Germany) and filtration through syringe filters (Rotilabo PP41 RC 0.20 µm, Carl Roth, Karlsruhe, Germany) into HPLC vials. The final volumes were stored at 4 °C until analysis.

### LC-MS/MS analysis

Ergosterol was analyzed by LC-MS/MS using a 1290 Infinity II HPLC system (Agilent, Waldbronn, Germany) coupled to a QTRAP 6500+ triple-quadrupole mass spectrometer (Sciex, Darmstadt, Germany). HPLC separation was performed on a ReproSil Gold 120 C18 column (100 mm × 2 mm, 3 µm; Dr. Maisch, Ammerbruch-Entringen, Germany) at a flow rate of 0.4 mL/min. Eluent A was water with 0.1 % (*v/v*) formic acid, eluent B consisted of methanol with 0.1 % (*v/v*) formic acid. The following gradient was used: 0 min, 20 % B; 1 min, 100 % B; 7 min, 100 % B; 7.5 min, 20 % B, 10 min, 20 % B. The injection volume was 10 µL and the column temperature was set to 40 °C. The mobile phase was diverted to waste from 0 to 3 min and from 7 to 10 min to keep contamination of the mass spectrometer to a minimum.

Atmospheric pressure chemical ionization in positive MRM mode (multiple reaction monitoring) was applied for detection using mass transition *m/z* 379 → 69 for quantification and mass transitions *m/z* 379 → 55 and *m/z* 379 → 41 for confirmation. The ion source temperature was set at 350 °C and a nebulizer current of 3 µA was applied. Nitrogen was used as curtain and collision gas, while air was used as nebulizer gas. The curtain gas was set at 35 psi, the nebulizer gas at 50 psi and the collision gas at medium. Further MS settings and compound specific parameters are summarized in Table 1.

**Table 1:** Compound specific parameters and MS-settings. The dehydrated molecular ion [M+H−H_2_O]^+^ was used as precursor ion. Retention time (RT), declustering potential (DP), entrance potential (EP), collision energy (CE), cell exit potential (CXP).

Compound	RT [min]	Precursor Ion [*m/z*]	Product Ion [*m/z*]	DP [V]	EP [V]	CE [V]	CXP [V]	Dwell Time [msec]
Ergosterol	4.52	379	69	66	10	49	12	100
		379	55	66	10	79	8	100
		379	41	66	10	93	6	100
7-Dehydrocholesterol	4.65	367	159	71	10	31	8	100
		367	145	71	10	29	12	100

### Calculation of results

Results are calculated based on peak areas (external calibration) or relative peak areas (internal calibration with internal standard). The calculation is described by Eq. (1) and (2):

(1) $$c_{c}=\;\frac{A\;-\;b}{m}$$

(2) $$\begin{array}{c} c_{S}\;=\;c_{c}\;\times \;\frac{V_{\text{End}}\;\times \;V_{\text{Ex}}}{W\;\times \;V_{A}} \end{array}$$

where, c_c_ is concentration of the analyte in the final extract calculated from the calibration curve in ng/mL, A is peak area in counts or relative peak area of the analyte, b is y-axis intercept of the calibration curve, m is slope of calibration curve, c_S_ is measured concentration of the analyte in the sample in µg/g, V_End_ is final volume in isopropanol in mL, V_Ex_ is volume of cyclohexane used for extraction in mL, W is initial sample weight in mg and V_A_ is aliquot volume of the cyclohexane extract in mL. An example calculation can be found in Annex A.

## Method validation

The validation was carried out according to residue analytical method guideline SANTE/2020/12830 [21] with respect to limit of detection and quantification, recovery and repeatability, matrix effects, stability of standard solutions, extract stability as well as freezer storage stability of samples. Furthermore, within-laboratory reproducibility and robustness of sample preparation with respect to sample weight and aliquot volume were investigated.

### Limit of detection and quantification

Environmental concentrations of ergosterol in aquatic leaf litter can range from about 10 to 1000 µg/g dw, where values around or even less than the lower limit hint at low fungal biomass, e.g. due to biological variance or unfavourable environmental conditions (e.g. [4,14]). However, according to our experience and literature, mean ergosterol concentrations are even in fungicide-treated leaves usually in the range of about 20 – 55 µg/g dw (e.g. [14,35,36]). Therefore, we aimed at a limit of quantification (LOQ) of 10 µg/g dw.

**Fig. 1:**
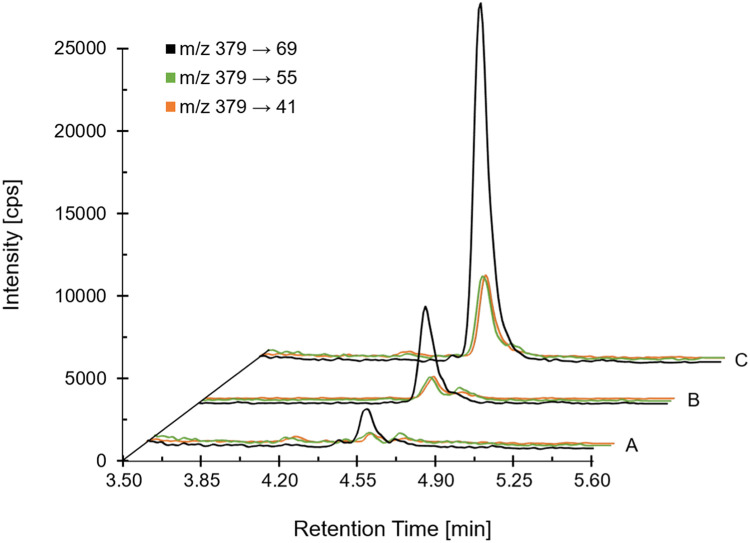
Extracted ion chromatograms for ergosterol of A) matrix blank with internal standard, B) lowest calibration standard (LOD) and C) recovery sample at LOQ. Matrix blank with internal standard: average measured concentration about 1.8 µg/g dw (equivalent to 18 % of LOQ), LOD: lowest calibration standard of 1.5 ng/mL (corresponding to 3 µg/g dw), LOQ: recovery sample at 10 µg/g dw (corresponding to 5 ng/mL).

Due to the presence of fungi on the collected alder leaves, a considerable ergosterol background between 3 µg/g dw and 11 µg/g dw was observed in freeze-dried blank sample material that was intended for the preparation of matrix spikes. Therefore, selected blank material was pooled and exposed to UV light (15 W lamp) for 96 h using an incubator hood (TH 30, Edmund Bühler, Bodelshausen, Germany) resulting in an average ergosterol content of about 1.8 µg/g dw (see Fig. 1 and Fig. S1). As a consequence, the limit of detection (LOD) of the method was set to 3 µg/g dw which is in accordance with the validation requirements of SANTE/2020/12830 [21] stating that concentrations in matrix blanks used for validation should not exceed 30 % of the LOQ.

Accordingly, the concentration of the lowest calibration standard was adapted to the LOD. Based on the sample preparation steps including the 1:10 reconstitution in the final evaporation step, a concentration of 1.5 ng/mL was calculated and applied (Fig. S3). The lowest fortification level was set to 10 µg/g dw and successfully validated with respect to recovery and repeatability. Nevertheless, if values lower than 10 µg/g dw are expected to occur within the chosen study design, a reduction of the LOQ/LOD is possible (see sections ‘*Sample preparation robustness*’ and ‘*Limitations and advantages*’).

**Fig. 2:**
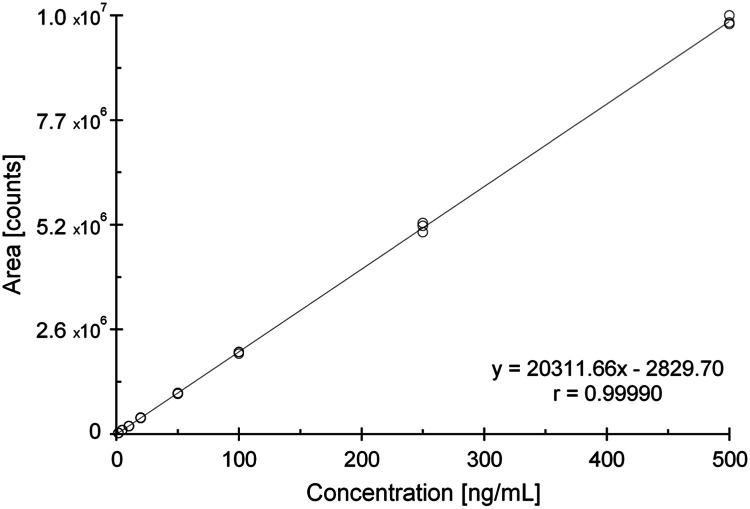
External calibration curve, linear equation and regression coefficient for ergosterol mass transition *m/z* 379 → 69. Each concentration level was injected three times.

### Recovery and repeatability

For recovery experiments, alder leaves were fortified at 10 µg/g dw (LOQ) and 500 µg/g dw. Repeatability of the analytical method was tested using five replicates per fortification level. Three mass transitions (*m/z* 379 → 69, 379 → 55 and 379 → 41) were evaluated for quantification and confirmation. Recoveries were determined by external calibration and blank value subtraction according to Eq. (3). For an example calculation, see Annex A.

(3) $$\text{Recovery}\;[\%]\;=\frac{c_{S}\;-\;\text{Blank}\;\text{Value}}{\text{Fortification}\;\text{Level}}\times 100$$

Mean recoveries ranging from 95.1 to 100.2 % at 10 µg/g dw and 95.4 to 96.7 % at 500 µg/g dw with relative standard deviations (RSD) < 10 % prove the validity of the method for each mass transition (see Table 2 and Tables S1 - S3 in the annex). Overall mean recoveries between 95.3 and 98.5 % were obtained, the overall RSD values were below 10 %. These results are in full agreement with the acceptance criteria of SANTE/2020/12830 [21] as mean recoveries are within 70 to 120 % and RSDs ≤ 20 %.

**Table 2:** Recovery data and relative standard deviations (RSDs) of validation samples determined by external calibration and blank subtraction.

Mass Transition	Fortification Level [µg/g dw]	n	Mean Recovery [%]	RSD [%]	Overall Mean Recovery [%]	Overall RSD [%]
379 → 69	10	5	100.2	7.7	98.5	8.2
	500	5	96.7	9.3		
379 → 55	10	5	95.1	9.2	95.3	8.7
	500	5	95.4	9.3		
379 → 41	10	5	99.4	9.6	97.6	8.9
	500	5	95.8	8.9		

### Selectivity and specificity

Although significant interferences (> 30 % of LOQ) were not detected at the retention time of ergosterol in matrix blanks after UV treatment, an interfering peak in the extracted ion chromatograms of ergosterol was observed that eluted shortly after ergosterol in samples containing the internal standard 7-dehydrocholesterol (see Fig. 1 and Fig. S2 – Fig. S4). Since this signal is not present in matrix blanks without internal standard (Fig. S1), the interference is likely an impurity of the internal standard. Depending on the mass transition considered, the intensity of the impurity is at a maximum of 50 % of the LOD for ergosterol but only at about 10 % for mass transition *m/z* 379 → 69 that is intended for quantification (see Fig. S3). In addition, the impurity is partially separated from ergosterol, allowing for the manual or automated integration of the ergosterol peak. Hence, the LC-MS/MS method with three mass transitions is suitable for the specific determination of ergosterol in alder leaves.

### Linearity

A linear external calibration with 1/x weighting and eight concentration levels ranging from 1.5 ng/mL (corresponding to the LOD of 3 µg/g dw) to 500 ng/mL was used (see Fig. 2). Calibration solutions were injected in triplicate. The concentration range corresponds to 3 - 1000 µg/g dw of ergosterol in leaf samples and covers more than two orders of magnitude. As a consequence, all recovery samples fitted within the calibration range with no need for dilution. The calibration functions for all mass transitions show correlation coefficients ≥ 0.999 (see Fig. 2, Fig. S5 and Fig. S6). In addition, the deviations of the back-calculated concentrations of the calibration standards are below ±20 % of the nominal concentrations (data not shown), fulfilling the requirements of guidance document SANTE/12682/2019 [37].

### Matrix effects

In order to evaluate the effect of the sample matrix on the intensity of the ergosterol signal, solvent-based standards were compared with matrix-matched standards. For this, the response factor of ergosterol (peak area divided by nominal concentration) was calculated for three concentration levels (5, 50 and 250 ng/mL, each injected in triplicate) of standards prepared in isopropanol and in matrix extract. Based on mean response factors, matrix effects were calculated for all mass transitions using Eq. (4). The matrix effects for all mass transitions (*m/z* 379 → 69, 379 → 55 and 379 → 41) ranged between −1.6 and 1.8 % (see Table 3), which is below the guideline threshold of ±20 % [21]. Therefore, solvent-based calibration standards were used for quantification. Nevertheless, potential matrix effects can be compensated by the use of 7-dehydrocholesterol as internal standard (see section ’*Internal standard*’).

(4) $$\text{Matrix}\;\text{Effect}\;[\%]=\frac{100\;\times \;\text{Mean}\;\text{Response}\;\text{Factor}\;(\text{Matrix})}{\text{Mean}\;\text{Response}\;\text{Factor}\;(\text{Solvent})}-\;100$$

**Table 3:** Assessment of matrix effects for mass transitions *m/z* 379 → 69, 379 → 55 and 379 → 41 by comparing mean response factors of standard solutions prepared in solvents and in blank matrix. The mean response factors were calculated from the mean values of three injections per concentration level.

			*m/z* 379 → 69		*m/z* 379 → 55		*m/z* 379 → 41	
Standard	Concentration Levels [ng/mL]	Injections per Level	Mean Response Factor	Matrix Effect [%]	Mean Response Factor	Matrix Effect [%]	Mean Response Factor	Matrix Effect [%]
Solvent	5^a^, 50^b^, 250^c^	3	6217	0.7	1477	−1.6	1323	1.8
Matrix	5^a^, 50^b^, 250^c^	3	6259		1453		1347	

a equivalent to LOQ.

b equivalent to 10-fold LOQ.

c equivalent to 50-fold LOQ.

### Sample preparation robustness

Two key aspects to increase the flexibility and working range of the method are initial sample weight and aliquot volume of the cyclohexane extract used for evaporation and re-dissolving. For method validation, 50 mg of homogenized leaf material and 100 µL aliquots were used. However, environmental sample material may vary in its quantity leading to sample weights < 50 mg. Thus, different initial weights and aliquot volumes were tested. For this purpose, two colonized alder leaf samples with different ergosterol content were homogenized. Three aliquots of 10, 50 and 100 mg were weighed from each sample and further processed as described above, but with varying volumes of cyclohexane aliquots (50, 100, 200 and 400 µl) including some exceptions due to the upper limit of the calibration range (see Table 4). With regard to relative standard deviations between 2.4 and 9.3 %, no substantial differences among all tested variations could be observed. In addition, the reliability of the applied sample homogenization technique could be demonstrated. If required, the LOQ of 10 µg/g dw could therefore be reduced up to a factor of four by keeping the initial weight at 50 mg and increasing the aliquot volume to 400 µL or weighing 100 mg of sample and using a 200 µL aliquot for further processing. If only a small amount of sample material is available, it is feasible to weigh in as low as 10 mg and use 400 µL sample extract for evaporation and re-dissolving without changing the LOQ.

**Table 4:** Results of sample preparation robustness test. Aliquots of samples A and B were analyzed using varying initial weights (10, 50 and 100 mg) and aliquot volumes of the cyclohexane extract (50, 100, 200 and 400 µL) used for evaporation and re-dissolving. RSD values < 10 % demonstrate the flexibility of the method with regard to initial weight and aliquot volume.

Sample	Initial Weight^a^ [mg]	Concentration [µg/g dw]					RSD [%]
		50 µL	100 µL	200 µL	400 µL	Mean	
A	10	126 ± 2.1	125 ± 5.1	128 ± 5.7	122 ± 1.3	125 ± 4.0^b^	3.1^b^
	50	118 ± 3.3	117 ± 3.6	117 ± 4.3	119 ± 4.0	118 ± 3.3^b^	2.7^b^
	100	116 ± 7.7	119 ± 4.6	119 ± 3.9	–	118 ± 5.2^c^	4.1^c^
B	10	232 ± 6.6	231 ± 5.6	230 ± 7.6	229 ± 6.3	230 ± 5.8^b^	2.4^b^
	50	223 ± 7.2	225 ± 4.6	229 ± 6.5	–	226 ± 5.9^c^	2.5^c^
	100	218 ± 11	220 ± 10	–	–	219 ± 10^d^	9.3^d^

a *n* = 3.

b *n* = 12.

c *n* = 9.

d *n* = 6.

### Internal standard

A suitable internal standard ensures effective compensation for matrix effects, volume errors and measurement deviations. In order to demonstrate the suitability of 7-dehydrocholesterol as internal standard, we compared recovery and repeatability data obtained by quantification without (Table 2) and with internal standard (Table 5). The mean and overall recoveries for each mass transition from internal calibration, ranging from 91.0 to 96.0 %, differ less than 5 % from the recoveries obtained by external calibration. No considerable differences were observed in the comparison of the relative standard deviations either. Internal calibration curves also show linearity with correlation coefficients ≥ 0.999 (see Fig. S7 - Fig. S9) and deviations of the back-calculated concentrations of the calibration standards below ±20 % of the nominal concentrations (data not shown). The excellent agreement of the validation results between internal and external calibration demonstrates that no crucial losses occur during sample preparation. The method provides valid results both with and without the use of an internal standard, which can therefore be seen as optional.

**Table 5:** Recovery data and relative standard deviations (RSDs) of validation samples determined by internal calibration and blank subtraction.

Mass Transition	Fortification Level [µg/g dw]	n	Mean Recovery [%]	RSD [%]	Overall Recovery [%]	Overall RSD [%]
379 → 69	10	5	96.0	4.9	94.7	7.1
	500	5	93.4	9.3		
379 → 55	10	5	91.0	6.6	91.6	7.7
	500	5	92.1	9.3		
379 → 41	10	5	95.2	7.6	93.9	7.9
	500	5	92.6	9.0		

### Within-laboratory reproducibility

During routine analysis, additional recovery data was obtained from ongoing method verification with samples fortified at 10 and 500 µg/g dw using mass transition *m/z* 379 → 69 for quantification. Concurrent recoveries at both concentration levels ranged from 86.4 to 110.6 % over a period of 149 days (see Table 6). The high and nearly identical precision at both fortification levels, as indicated by relative standard deviations of 7.6 and 8.7 %, demonstrates a good within-laboratory reproducibility of the method.

**Table 6:** Ongoing method verification data of ergosterol in leaves over a period of 149 days using mass transition *m/z* 379 → 69 for quantification. RSD_wR_ values below 10 % demonstrate a good within-laboratory reproducibility (w*R*).

Fortification Level [µg/g dw]	n	Mean Recovery [%]	Min Recovery [%]	Max Recovery [%]	RSD_wR_ [%]
10	8	99.0	87.1	108.4	8.7
500	8	99.1	86.4	110.6	7.6

### Storage stability

For the investigation of the freezer storage stability, four colonized alder leaf samples were pooled and prepared as described above. The pooled sample was reanalyzed after storage for 139, 167, 319 and 474 days at −20 °C in the dark. The ergosterol content obtained from initial analysis of the sample was assumed to be 100 %. Based on the determined recovery values between 104 and 114 % and a relative standard deviation of 4.3 % (data not shown), the storage stability of ergosterol in alder leaves was verified for at least 474 days under the abovementioned conditions.

### Stability of extracts and standard solutions

The stability of ergosterol in the cyclohexane extract and in the final volume in isopropanol was tested at concentrations corresponding to 10 µg/g dw (LOQ) and 500 µg/g dw using samples from method validation. The initial values were set as 100 %. After storage for 7 days at 4 °C in the dark, the extracts and final volumes were reanalyzed. The mean recovery values at both fortification levels ranged between 100 and 109 % for reprocessed sample extracts and 101 to 107 % (*n* = 3 each) for remeasured final volumes (data not shown). Hence, ergosterol is stable in cyclohexane extracts and final volumes in isopropanol for at least 7 days when stored refrigerated.

Stock and calibration solutions of ergosterol in isopropanol were stored at 4 °C in the dark and compared to freshly prepared solutions. Stock solutions were diluted with isopropanol to 0.25 mg/L and analyzed after 5, 85, 112 and 181 days of storage. Calibration solutions with ergosterol concentrations of 5, 50 and 500 ng/mL were measured undiluted after 8 and 112 days. Mean area values of at least three injections of stored and freshly prepared solutions were compared. The mean values of all compared solutions differed not more than 10 % at all investigated time points demonstrating the stability of ergosterol under the given conditions for 181 days in stock solutions and for 112 days in calibration solutions.

## Limitations and advantages

The current method is based on the selectivity and sensitivity provided by LC-MS/MS. As a consequence, the method does not require sample enrichment in comparison to the original textbook method by Gessner using SPE and HPLC-UV [4]. Here, the sample extract is enriched via SPE by a factor of at least 6.25, whereas the current method relies on a 1:10 reconstitution of the dried extract before injection. Nevertheless, if no LC-MS/MS system is available, HPLC-UV can as well be used for detection as also described by Headley et al. [16], Verma et al. [18] and Mille-Lindblom and Tranvik [20]. However, care has to be taken with regard to false positive results [16,18].

Instead of SPE we use LLE for sample clean-up. Gessner and Schmitt [5] could show that SPE and LLE lead to similar results when samples are analyzed using both extraction techniques. Based on our experience, LLE is less time consuming and error-prone than SPE, especially since our method requires only a single extraction step instead of multiple extractions. Verma et al. [18] tested different extraction conditions and discovered that KOH in methanol followed by LLE with pentane results in clean sample extracts due to the separation of polar compounds by LLE and a high extraction efficiency. We used cyclohexane for the extraction of ergosterol from alkaline methanol because of its lower toxicity compared to pentane [34]. For heat-reflux extraction, we utilized semi-closed digestion units made of PFA instead of closed glassware vessels as in the above-mentioned publications. The use of an open extraction system avoids working under high pressure and thus enhances lab safety.

Since the method was validated for leaf samples, the applicability of the method to other matrices has to be proven. In another study of ours (in preparation), the suitability for fungal biomass samples could already be demonstrated. The use of 7-dehydrocholesterol as internal standard to compensate signal drift is optional and depends on the mass spectrometer used. Although the signal response was stable with the LC-MS/MS used for the present method validation, a signal drift in mass spectrometric ionization response with another LC-MS/MS instrument was observed during the ongoing analysis of samples. This drift could be successfully compensated with 7-deyhdrochloesterol. The sensitivity of the method, i.e. the LOD of 3 µg/g dw and the LOQ of 10 µg/g dw, was adapted to ergosterol background levels of about 1.8 µg/g dw in alder leaves previously exposed to UV light in order to reduce the ergosterol content caused by leaf associated fungi (e.g. plant pathogens, epi‑ and endophytes). According to our experiences, the ergosterol background can even in fresh leaves be as high as 11 µg/g dw and cannot be reduced by autoclaving the leaf material. If blanks of other matrices contain less ergosterol, the sensitivity can be adapted accordingly by increasing the sample weight, the aliquot volume used for evaporation, the volume of isopropanol used for reconstitution of the residue and/or the injection volume, respectively.

## Ethics statements

Not applicable.

## CRediT authorship contribution statement

**Björn Kusebauch:** Conceptualization, Methodology, Visualization, Writing – original draft, Writing – review & editing, Resources, Supervision. **Stefan Bader:** Methodology, Investigation, Validation, Formal analysis, Data curation, Visualization, Writing – original draft, Writing – review & editing. **Steffen Carl:** Conceptualization, Methodology, Investigation, Writing – original draft, Writing – review & editing, Project administration, Resources.

## Declaration of competing interest

The authors declare that they have no known competing financial interests or personal relationships that could have appeared to influence the work reported in this paper.

## Data Availability

Data will be made available on request.

## Appendix Supplementary materials

Image, application 1
