# Supplementary material for: Development and validation of an alternative method for ergosterol determination in alder leaves using liquid-liquid extraction and LC-MS/MS after saponification
Source: MethodsX. 2025 Jul 7;15:103495. doi: 10.1016/j.mex.2025.103495 (PMC12301759; doi:10.1016/j.mex.2025.103495)
Supplement: Supplementary file 1 [file mmc1.pdf]

Supplementary Material

Development and validation of an alternative method for ergosterol determination in alder leaves using liquid-liquid extraction and LC-MS/MS after saponification

Björn Kusebauch*, Stefan Bader, Steffen Carl

German Environment Agency (UBA), Section IV 2.5 - Trace Analysis, Artificial Ponds and Streams, Schichauweg 58, 12307 Berlin, Germany

**corresponding author (bjoern.kusebauch@uba.de;phone: +4930 8903 4147)*

Annex A: Example for the calculation of results

An example calculation for a recovery sample fortified at 10 µg/g dw is shown in Table A.2. Mass transition *m/z* 379 → 69 was used for quantification and results were calculated according to Eq. (1-3) with values listed in Table A.1.

$$c_C = \frac{A - b}{m} \quad (1)$$

$$c_S = c_C \times \frac{V_{End} \times V_{Ex}}{W \times V_A} \quad (2)$$

$$\text{Recovery [\%]} = \frac{c_S - \text{Blank Value}}{\text{Fortification Level}} \times 100 \quad (3)$$

Table A.1
Values used for calculation

Parameter	Value
Peak area A (mean of two injections)	110114.7 counts
Intercept b	- 2829.7 counts
Slope m	20311.7 counts × mL/ng
Initial sample weight	50 mg
Volume of cyclohexane used for extraction V_{EX}	10 mL
Aliquot volume of cyclohexane extract V_A	0.1 mL
Final volume V_{End}	1 mL
Ergosterol content in matrix blank	1.79 µg/g dw

Table A.2
Calculation of results

Parameter	Calculation	Result
Concentration in the final extract c_c	$\frac{110114.7 \text{ counts} + 2829.7 \text{ counts}}{20311.7 \text{ counts*mL/ng}}$	5.56 ng/mL
Measured concentration in the sample c_S	$5.56 \text{ ng/mL} \times \frac{1 \text{ mL} \times 10 \text{ mL}}{50 \text{ mg} \times 0.1 \text{ mL}}$	11.12 µg/g dw
Final concentration after blank value subtraction	11.12 µg/g dw - 1.79 µg/g dw	9.33 µg/g dw
Recovery	$\frac{9.33 \text{ µg/g dw}}{10 \text{ µg/g dw}} \times 100$	93.3 %

Supplementary tables and figures

Table S1
Method validation data of ergosterol in leaves for mass transition *m/z* 379 → 69 determined by external calibration and blank subtraction.

Fortification Level [µg/g dw]	Concentration after blank subtraction [µg/g dw]	Recovery [%]	Mean Recovery [%]	RSD [%]
10	9.33	93.3	100.2	7.7
10	9.15	91.5		
10	10.1	101.0		
10	10.7	107.4		
10	10.8	107.9		
500	473	94.7	96.7	9.3
500	562	112.4		
500	472	94.5		
500	449	89.8		
500	462	92.4		

Table S2
Method validation data of ergosterol in leaves for mass transition *m/z* 379 → 55 determined by external calibration and blank subtraction.

Fortification Level [µg/g dw]	Concentration after blank subtraction [µg/g dw]	Recovery [%]	Mean Recovery [%]	RSD [%]
10	8.78	87.8		
10	8.67	86.7		
10	9.33	93.3	95.1	9.2
10	10.0	100.2		
10	10.7	107.5		
500	466	93.2		
500	555	111.0		
500	463	92.5	95.4	9.3
500	444	88.8		
500	457	91.5		

Table S3
Method validation data of ergosterol in leaves for mass transition *m/z* 379 → 41 determined by external calibration and blank subtraction.

Fortification Level [µg/g dw]	Concentration after blank subtraction [µg/g dw]	Recovery [%]	Mean Recovery [%]	RSD [%]
10	9.33	93.3		
10	8.91	89.1		
10	9.65	96.5	99.4	9.6
10	10.6	105.7		
10	11.2	112.5		
500	478	94.3		
500	526	110.7		
500	446	93.5	95.8	8.9
500	445	88.9		
500	418	91.8		

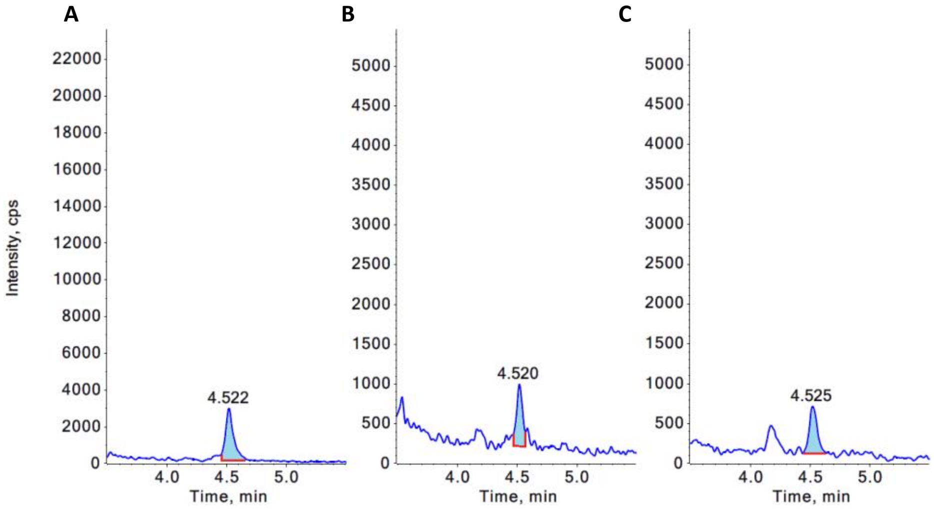

Fig. S1. Chromatograms of matrix blank without internal standard, measured concentration < LOD (3 µg/g dw). A: ergosterol *m/z* 379 → 69. B: ergosterol *m/z* 379 → 55. C: ergosterol *m/z* and 379 → 41.

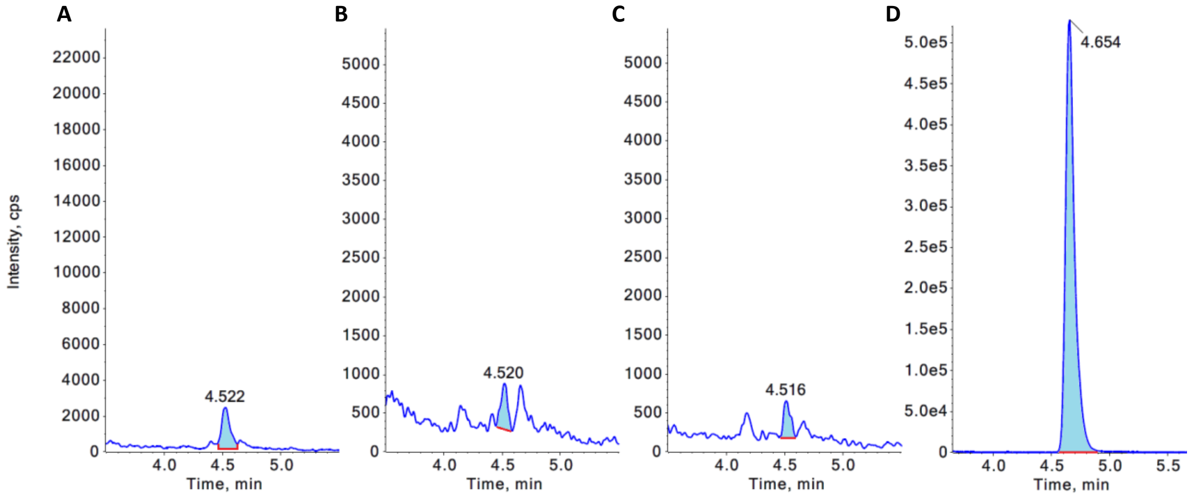

Fig. S2. Chromatograms of matrix blank with internal standard, measured concentration < LOD (3 µg/g dw). A: ergosterol *m/z* 379 → 69. B: ergosterol *m/z* 379 → 55. C: ergosterol *m/z* 379 → 41. D: 7-dehydrocholesterol *m/z* 367 → 159, c = 100 ng/mL.

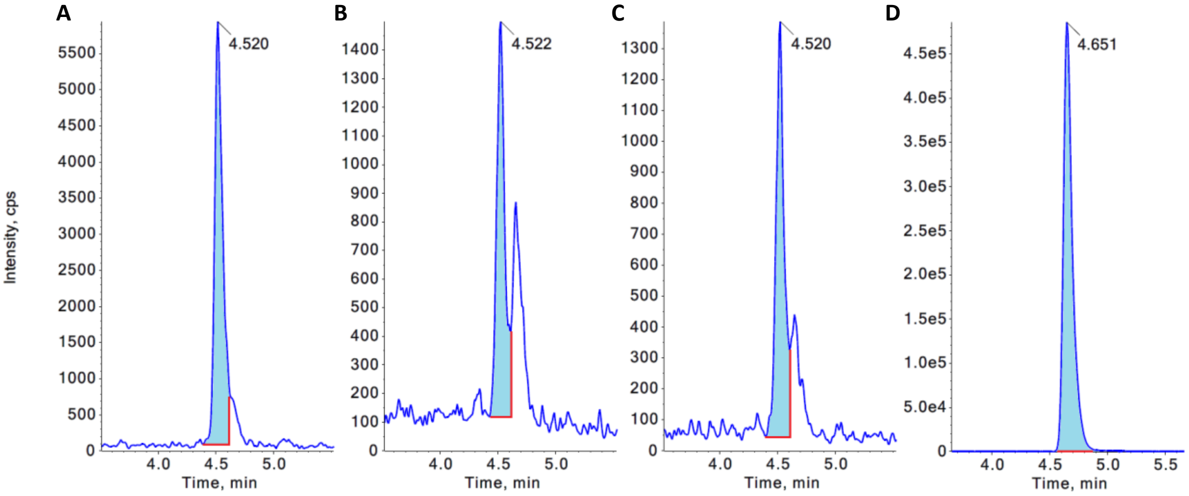

Fig. S3. Chromatograms of lowest calibration standard corresponding to LOD (c = 1.5 ng/mL, equivalent to 3 µg/g dw in leaves). A: ergosterol *m/z* 379 → 69. B: ergosterol *m/z* 379 → 55. C: ergosterol *m/z* 379 → 41. D: 7-dehydrocholesterol *m/z* 367 → 159, c = 100 ng/mL.

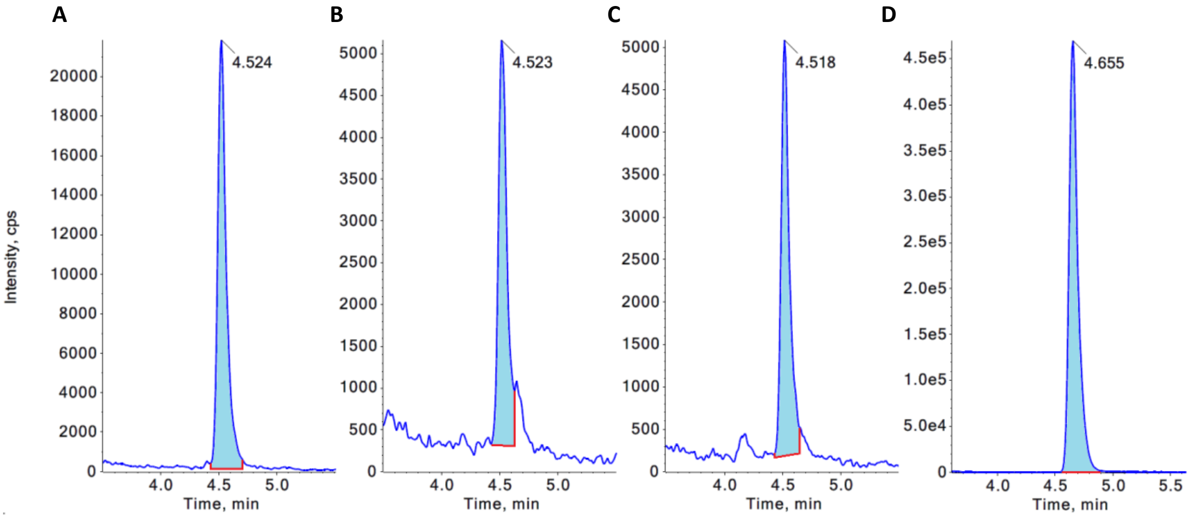

Fig. S4. Chromatograms of matrix blank fortified with ergosterol at 10 µg/g dw (LOQ), recovery 93.3 % (*m/z* 379 → 69). A: ergosterol *m/z* 379 → 69. B: ergosterol *m/z* 379 → 55. C: ergosterol *m/z* 379 → 41. D: 7-dehydrocholesterol *m/z* 367 → 159, c = 100 ng/mL.

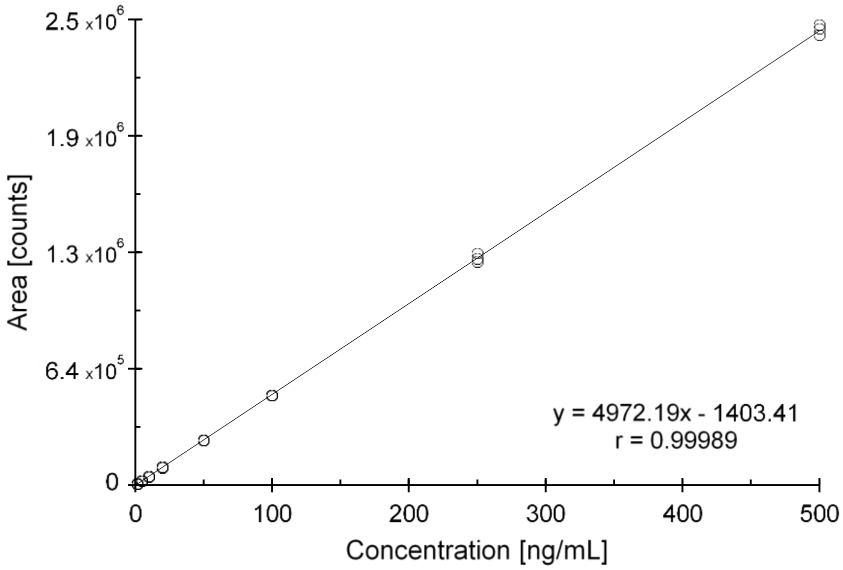

Fig. S5. External calibration curve. linear equation and regression coefficient for ergosterol mass transition *m/z* 379 → 55. Each concentration level was injected three times.

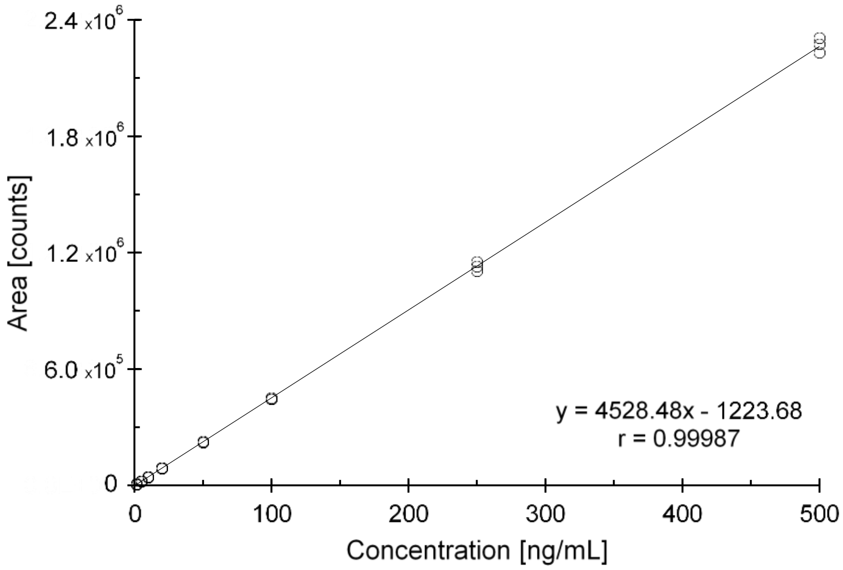

Fig. S6. External calibration linear, equation and regression coefficient for ergosterol mass transition *m/z* 379 → 41. Each concentration level was injected three times.

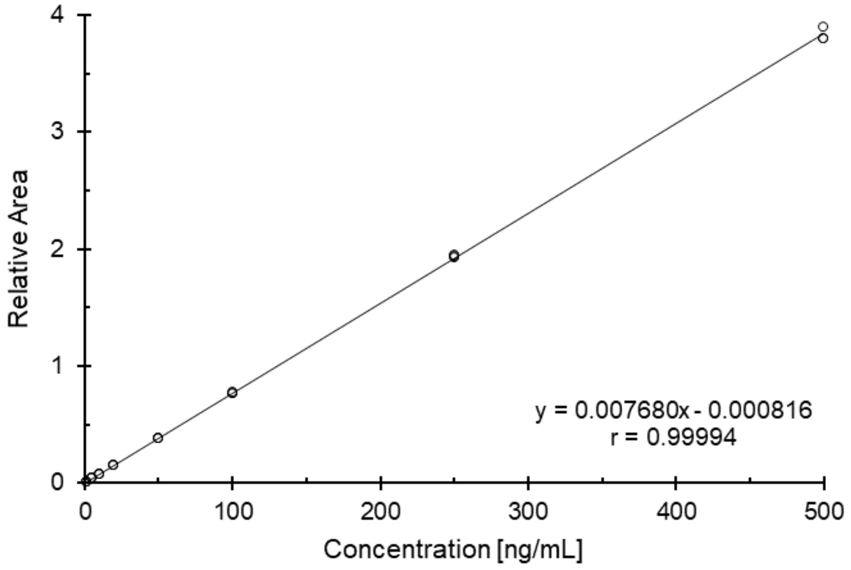

Fig. S7. Internal calibration curve, linear equation and regression coefficient for ergosterol mass transition *m/z* 379 → 69 using 7-dehydrocholesterol as internal standard. Each concentration level was injected three times.

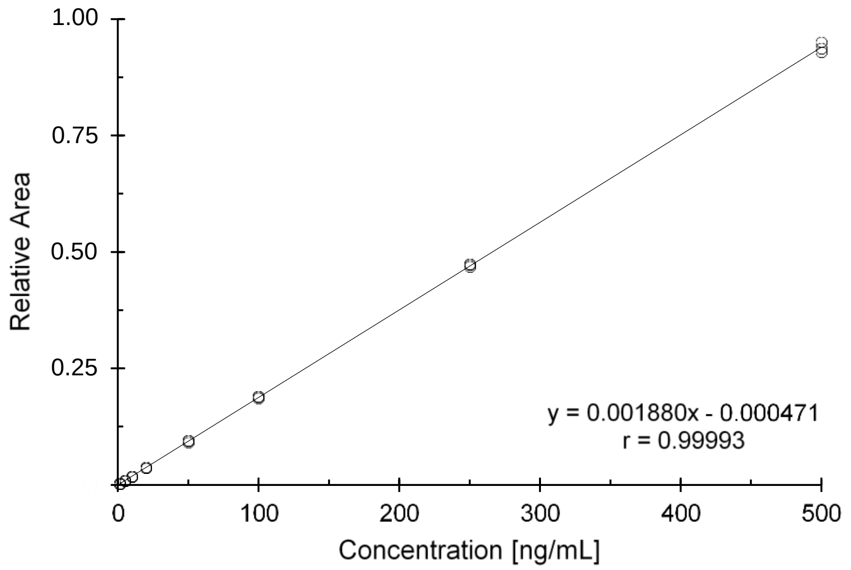

Fig. S8. Internal calibration curve, linear equation and regression coefficient for ergosterol mass transition *m/z* 379 → 55 using 7-dehydrocholesterol as internal standard. Each concentration level was injected three times.

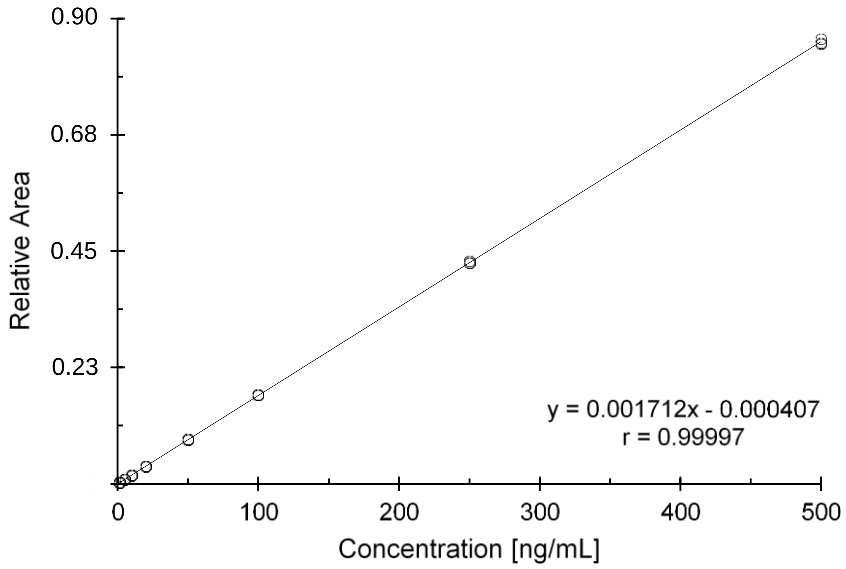

Fig. S9. Internal calibration curve, linear equation and regression coefficient for ergosterol mass transition *m/z* 379 → 41 using 7-dehydrocholesterol as internal standard. Each concentration level was injected three times.
